# Effects of Asymmetric Quantum Wells on the Structural and Optical Properties of InGaN-Based Light-Emitting Diodes

**DOI:** 10.3390/ma7053758

**Published:** 2014-05-12

**Authors:** Chia-Lung Tsai, Wei-Che Wu

**Affiliations:** Department of Electronic Engineering and Green Technology Research Center, Chang Gung University, Taoyuan 333, Taiwan; E-Mail: t0924000886@hotmail.com

**Keywords:** InGaN, asymmetric quantum well (AQW), light-emitting diodes (LEDs)

## Abstract

A metalorganic vapor phase epitaxy-grown InGaN/GaN multiple-quantum-well (MQW) with three graded-thickness wells (the first-grown well had the greatest width) near the n-GaN was used as the active layer of an LED. For LEDs with an asymmetric quantum well (AQW), high-resolution X-ray diffraction and transmission electron microscopic reveal that the modified MQWs with a reasonable crystalline quality were coherently strained on the underlying GaN epilayers without any relaxation. In addition, the slight increase of indium segregation in the LED with an AQW may be attributed to variations in indium contents experienced during epitaxial growth of the wide well-containing MQWs. By preventing the energetic electrons from accumulating at the topmost quantum well nearest the p-GaN, the presence of light intensity roll-off in the LED with an AQW is shifted to higher currents and the corresponding maximum light output power is increased with a ratio 7.9% higher than that of normal LEDs. Finally, similar emission wavelengths were observed in the electroluminescence spectra of both LEDs, suggesting that light emitted mostly from the top quantum wells (near the p-GaN) while the emissions from the AQW region were insignificant.

## 1. Introduction

Solid-state lighting (SSL) based upon the brightness, efficiency, and long-term reliability of light-emitting diodes (LEDs) is extremely suitable for use in a wide variety of monochrome applications such as displays, traffic signals, and biological sensors [1]. In addition, white-light LEDs have been considered to be a promising candidate to replace conventional fluorescent lamps for general illumination [2]. Considering manufacturing costs and energy savings, further improvements to LED efficiency is critical to promoting the expanded use of LED luminaires. Generally, an InGaN/GaN multiple-quantum-well (MQW) is used as the active layer of visible LEDs with emission wavelengths in the near ultraviolet to green range. Although the as-grown InGaN LEDs have a high defect density (e.g., threading dislocations and stacking faults), the injection carriers can recombine radiatively at deep localized states associated with the formation of indium-rich nanoclusters (quantum-dot-like structures) in InGaN wells due to phase separation or spinodal decomposition, and contributes to strong luminescence [3]. In addition, InGaN MQWs feature a huge built-in electric field (~MV/cm) induced by the hexagonal symmetry of III-nitrides and the lattice mismatch between the InGaN and GaN epilayers, which will lead to significant deformation in the potential profile of rectangular-shaped wells. The quantum-confined Stark effect (QCSE) shifts the emission spectrum towards longer wavelengths, causing these LEDs to exhibit a low quantum efficiency due to decreased electron and hole wave functions overlapping within tilted potential wells [4]. Experimental results found that the degree of compositional inhomogeneity in indium content and the residual strain stored in InGaN is highly correlated with growth conditions [5–7]. Using an adequate strategy for the design of the MQWs (*i.e.*, modifying the barrier height of the heterojunctions, and the width of the well and barrier layers, *etc.*) makes it feasible to realize an InGaN LED with improved quantum efficiency. Zhao *et al.* [8] reported that the strain energy and the misfit-induced defects in InGaN can be reduced by using a strain-compensated InGaN/AlGaN MQW. Numerical simulations indicate that these LEDs with tensile AlGaN barrier layers not only exhibit improved spontaneous emission rates but also provide better carrier confinements in the quantum wells. Instead of using an AlGaN electron blocking layer, InGaN LEDs fabricated with an AlGaN/GaN multiquantum barrier (MQB) were investigated numerically [9]. The improved performance of the InGaN LEDs with a MQB may be due to energy band-bending at the last GaN barrier/MQB interface, thus increasing the effective barrier height for the electrons while decreasing that for the holes. The phenomenon of efficiency droop (nonthermal rollover), which is commonly observed in InGaN LEDs at a high current density, could be effectively addressed by the use of a wide well as light emitting medium [10,11]. This is attributed to the decreased carrier density in the wide wells, which helps to suppress nonradiative Auger recombination and reduce carrier leakage during the LED operation. Furthermore, Ni *et al.* [12] reported that coupled quantum wells consisting of a thin barrier can help increase the uniformity of hole distribution among the InGaN MQWs. As a result, the fabricated LEDs exhibit improved efficiency droop with a high crossover current density of up to 1100 A/cm^2^ under pulsed mode operations. On the other hand, the asymmetric MQW containing structures can also be utilized to improve the hole transport over the MQW regions, which is attributed to the distinct carrier capture efficiency of the quantum wells with different widths [13,14]. By using an indium composition graded well to achieve better overlap of the electron and hole wave functions in InGaN wells, the proposed LEDs with the asymmetrically graded wells exhibits an enhanced spontaneous emission rate compared to that of normal LEDs with rectangular wells [15].

This article describes the incorporation of an asymmetric quantum well (AQW) into the active layers of InGaN LEDs by metalorganic vapor phase epitaxy (MOVPE). Three wells with a graded thickness and located close to the n-side layers were used to improve carrier distribution within the quantum wells responsible for light emission. Furthermore, the effects of the AQW on the structural and optical properties of InGaN LEDs are characterized experimentally.

## 2. Results and Discussion

**Figure 1.**
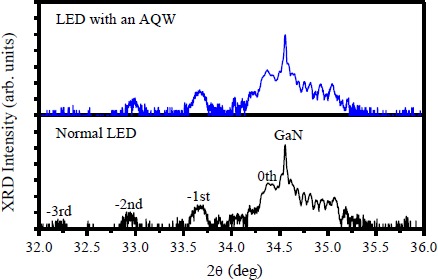
High-resolution X-ray diffraction (HRXRD) *w*-2θ scans of the (0002) reflections for the LEDs with and without an AQW.

Figure 1 displays the HRXRD *w*-2θ scans of the (0002) reflection for the LEDs with and without an AQW. The sharp peak at an angle of 34.56° comes from the diffraction of the n-GaN underlying layer. For both LEDs, several well-resolved satellite peaks (up to third order) can be clearly distinguished in the X-ray diffraction patterns, indicating that the crystalline perfection of epitaxial layers is well preserved even though part of the quantum wells adjacent to the n-GaN were replaced by the graded-thickness wells. Furthermore, the high contrast between the InGaN and GaN layers observed from the HRTEM image (shown in Figure 6) provides objective evidence that the sharp interface is not destroyed in the LEDs fabricated with an AQW-containing structure. Based upon the HRXRD *w*-2θ scans of the GaN (0002) and GaN (10 $\bar{1}$2) reflections, the MQW period and the average indium composition is estimated to be about 12.9 nm and 3.1%, respectively, for the normal LED [16]. In contrast, it is difficult to evaluate the structural parameters of the LED with an AQW due to the complexity of its epitaxial structures. The indium content in the wide wells may deviate from the others due to the composition pulling effect [17]. However, experimentally, both LEDs exhibited similar emission wavelengths over the entire range of current injections, as shown in Figure 5, suggesting that the LED with an AQW should have a similar indium composition at the top quantum wells (near the p-GaN) since the growth conditions (aside from the varied deposition time in the wide wells) were kept identical for both LEDs. HRTEM analysis also confirms the well and barrier thickness of the proposed LEDs meet our expectations.

**Figure 2.**
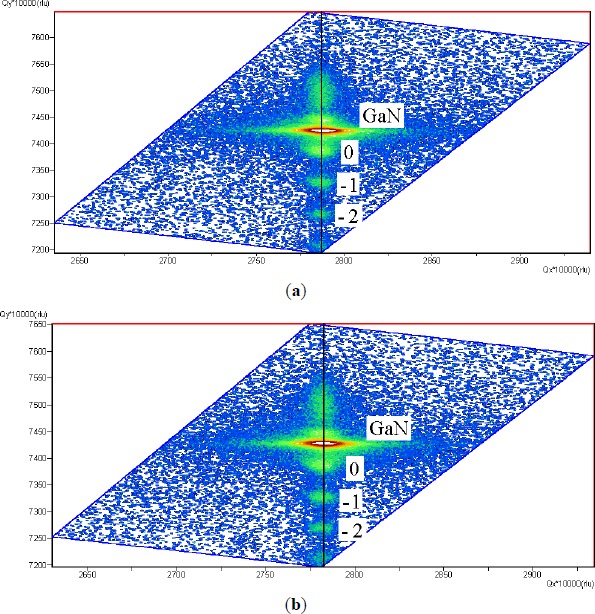
Reciprocal space mapping around the asymmetric (10 $\bar{1}$5) reflection for (**a**) normal LED; and (**b**) LED with an AQW.

Because InGaN MQWs were grown on lattice-mismatched GaN barriers, the formation of the misfit-induced defects (such as dislocations) through the partial relaxation of accumulated strain in InGaN will significantly affect the structural and optical properties of the fabricated LEDs [18]. Figure 2 shows reciprocal space mapping around the asymmetric (10 $\bar{1}$5) reflection for the normal LED and the LED with an AQW. The reciprocal lattice point of the GaN layer (upper side) and the satellite diffraction peaks of InGaN MQWs (lower side) are clearly on the same vertical line, implying that both LEDs were grown pseudomorphically on GaN without any relaxation along the in-plane direction.

**Figure 3.**
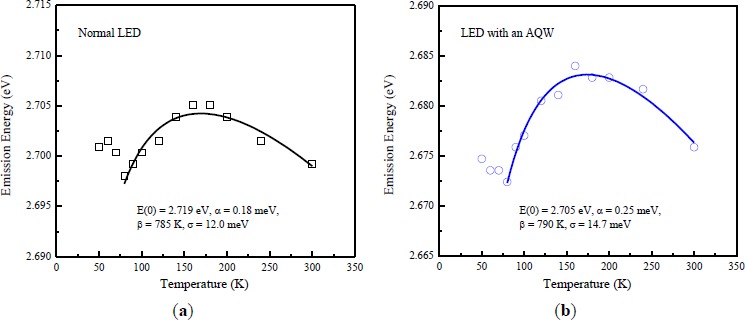
Temperature dependence of photoluminescence (PL) emission energy for (**a**) normal LED, and (**b**) LED with an AQW. The solid lines are fitted curves based on the band-tail model. The resulting fit parameters: *E*(0), α, β, and σ are 2.705 eV, 0.25 meV, 790 K, 14.7 meV, and 2.719 eV, 0.18 meV, 785 K, 12.0 meV, respectively, for the LEDs with and without an AQW.

Figure 3 shows photoluminescence (PL) emission energy as a function of temperature for the LEDs with and without an AQW. The presence of the “S-shaped” temperature dependence of the PL peaks was observed in both LEDs, implying a localized state in the band tails due to the occurrence of indium compositional fluctuation during InGaN growth [19]. As a result of increased carrier lifetime at very low temperatures, the photogenerated carriers have more opportunities to relax down to deeper localized states for effective radiative recombination and emitting with longer peak wavelengths as operating temperature increased. In addition, the PL emission peak starts move towards shorter wavelengths as temperature increased. This could be due to the decrease in carrier lifetime so that these carriers do not have enough time to reach the lower energy states and thus the higher energy side emission due to the thermal population of higher energy states was enhanced instead. Finally, temperature-induced bandgap shrinkage becomes dominant and responsible for the redshift behavior of the PL emission peak as the temperature increased to 300 K. Based on the band-tail model [20], the temperature-dependent emission energy curves could be fitted by the following expression to obtain the degree of exciton localization effect:

$$E(T)=E(0)-\frac{\alpha {Τ}^{2}}{T+\beta }-\frac{\sigma^{2}}{K_{B}T}$$ (1)

where *E*(0) is the energy gap at 0 K, α and β are known as Varshni’s fitting parameters, *k*_B_*T* is the thermal energy, and σ indicates the degree of the localization effect. Figure 3a,b clearly show that the localization effect is slightly strong in the LED with an AQW due to its larger value of σ (14.7 meV). Variations in material defects, indium content, well width and interface roughness between the well and barrier layers during epitaxial growth of InGaN MQWs all contribute to the formation of different levels of the localization effect [5–7,21–24]. As indicated in the discussion of the results of Figure 2, in both LEDs, InGaN MQWs were fully strained on GaN; therefore, partial strain relaxation through the process of spinodal decomposition may not be the main cause of the increased indium segregation in the LED with an AQW. In addition, high-magnification HRTEM image (not shown here) also indicates the high quality of interfaces and thickness uniformity of the LED with an AQW. In the experiment, the operation voltage is set to 200 kV during the HRTEM analysis of a high quality GaN sample prepared using a focused ion beam. Hao *et al.* [17] reported that indium atoms trend away from the InGaN/GaN interface to reduce the deformation energy owing to the lattice mismatch. For the LED with an AQW, the amount of indium could increase within a certain range along the growth direction of the wide wells (near the n-GaN) since the impact of the composition pulling effect becomes discernible with the use of a wide well. Consequently, the variation of indium contents in the AQW region may be responsible for the slight increase in the carrier localization degree of the LED with an AQW.

**Figure 4.**
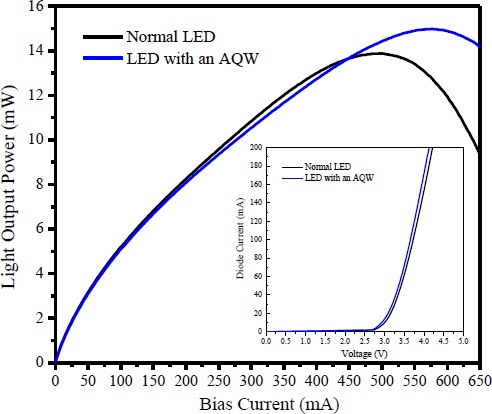
Light output power as a function of injection current for the LEDs with and without an AQW at 300 K. The inset shows the current-voltage (I-V) characteristics of the fabricated InGaN LEDs.

Figure 4 shows the light output power as a function of injection current measured at room temperature for both the normal LED and the LED with an AQW. As evaluated from the current-voltage characteristics (shown in the inset of Figure 4), the effective series resistance and the forward voltage are approximately 4 Ω and 3.1 V at 20 mA for both LEDs. This indicates that the incorporation of an AQW within the LED structures has little influence on the electrical properties of the fabricated LEDs. Inspection of Figure 4 reveals that, at low current levels, the light intensity increases with injection current for both LEDs because the spontaneous emission rate is proportional to the injection current density. In addition, both LEDs exhibited similar light intensity at an injection current below 200 mA. Previous studies of the efficiency droop effect in InGaN LEDs have found that carrier localization in the quantum-dot-like indium-rich regions helps these LEDs achieve a higher efficiency at low current densities, while the efficiency droop by the delocalization of carriers and then by the carrier leakage becomes severe in LEDs operated at elevated current levels [25]. Experimentally, such a result implies that even the injected carriers can recombine radiatively at a local potential minima caused by indium compositional inhomogeneities at a low current level. However, the exciton localization effect in the LEDs with an AQW is not sufficiently strong with respect to the normal LEDs to significantly improve their light intensity. Due to an increase in the degree of the carrier delocalization effect, the probability of the injection carriers being captured by the nonradiative recombination centers due to the existence of the material defects (e.g., threading dislocations) in InGaN MQWs will also increase with the injection current. This may degrade the quantum efficiency of both LEDs beginning at a low current (~100 mA) [25]. In addition, the LED with an AQW shows worse power than the device with a normal MQW as the injection current is increased from 200 mA up to 450 mA. Chen *et al.* [26] reported that a self-consistent APSYS simulation program can be used to theoretically investigate the internal physical processes within the InGaN MQWs, such as energy band diagram, carrier distribution, and radiative recombination rate, *etc.* In a five-pair In_0.2_Ga_0.8_N/GaN MQW containing LEDs, they found that the injected carriers prefer to concentrate at the quantum well closest to the p-side GaN at relatively low currents. Despite the holes’ large effective mass and small mobility relative to the electrons hindering their transport capacity, more quantum wells are indeed filled with some holes and can effectively participate in the radiative recombination process as LEDs operating at elevated current levels. Therefore, we speculate that, in the LEDs with normal MQW, more quantum wells (near the n-GaN) could also be filled with some amount of the holes under elevated current levels and then the holes will recombine radiatively with the electrons to contribute to light intensity. However, a similar situation may not be observed in the LED with an AQW because the QCSE is relatively strong in the wide wells, which will result in a reduced spontaneous emission rate in these wells. Further increasing the injection current will degrade the emission efficiency of the LEDs, which could be resulted from Auger nonradiative recombination or insufficient potential barrier height (provided a polarization unmatched AlGaN EBL was used) to confine carriers (electrons) within the InGaN MQWs [27,28]. At high levels of current injection, LED performance could also be deteriorated by increased junction temperature due to the increased probability of thermal carriers escaping from the MQWs [29]. Interestingly, the presence of the output power roll-off in the light intensity-current curve occurred at higher levels of injection current for the LED with an AQW than in the normal LED. The corresponding maximum light output power is evaluated as 14.97 mW at 580 mA and 13.88 mW at 500 mA, respectively, for the LEDs with and without an AQW. At high current levels, the emission properties of InGaN LEDs will be dominated by a band-to-band transition. This is due to the band filling of almost localized states in indium-rich areas with increasing current so that the subsequent injection of carriers will be released to the conduction band. Due to the quantum confinement effect, the ground-state energy level of electrons in the wide wells is lower than that in the narrow wells. For the LED with an AQW, the electrons injected from the n-GaN to the AQW will populate at the first-grown well with widest width because the difference in the ground-state energy between the quantum wells with different thicknesses will obstruct electrons tunneling into the narrow ones [13,30,31]. In addition, carrier collection capability was also improved by using a wide well with a higher density of allowed state. Both effects will alleviate the degree to which injected carriers (electrons) prefer to accumulate at the topmost quantum nearest the p-side GaN [32], which is commonly considered to be the probable cause of efficiency droop in InGaN LEDs. Thereby, better uniformity of carrier distribution within the quantum wells at high current density (especially for the top quantum wells near the p-GaN) is believed to be responsible for the results of improved light output performance in the LED with an AQW, as compared to that of the normal LED.

**Figure 5.**
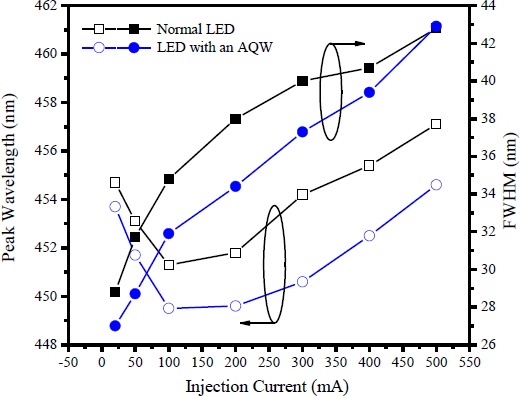
Dependence of the peak emission wavelength and the emission peak linewidth FWHM on injection current for the LEDs with and without an AQW.

Figure 5 shows the dependence of the peak emission wavelength and the emission peak linewidth’s full width at half-maximum (FWHM) on the injection current for the LEDs with and without an AQW. For both LEDs, free carrier screening of the strain-induced piezoelectric fields or band filling of the localized states are responsible for the observed blueshift behavior of the emission peaks in the injection current range from 20 to 100 mA [33], while the current-induced temperature effect contributes to a redshift in the emission peak wavelength of the electroluminescence (EL) spectrum at current levels above 100 mA [34]. In addition, the broadening of the EL emissions with increasing current is seen as being due to band filling effect [35]. Higher current injection levels were associated with the increased possibility of higher energy states being occupied with injected carriers. However, because most of these carriers prefer to relax down to the ground states, the ground-state recombinations will dominate the EL emission while only a small portion of light comes from higher energy emissions. As evaluated from the EL spectrum (not shown here) of the LED with an AQW, the amount of light (λ > 500 nm) emitted from the wide wells is trivial. Besides, as the injection current increased from 100 to 500 mA, the EL spectrum for the LED with an AQW exhibits a redshift of 5.1 nm and broadening of about 11 nm, compared to 5.8 and 8 nm, respectively, for the normal LED. In comparison with other studies using an all graded-thickness well to act as the active layer of InGaN LEDs [30,36], multicolor emissions can be suppressed in our proposed LEDs as part of the quantum wells near the n-GaN was grown with a graded width. Such results suggest that the amount of carrier recombination in the wide wells should be small because holes with a large effective mass and a low mobility can not penetrate significantly deeper into the quantum wells [12]. In addition, the large internal field caused by the strong QCSE in these wells slows the radiative recombination rate.

## 3. Experimental Section

**Figure 6.**
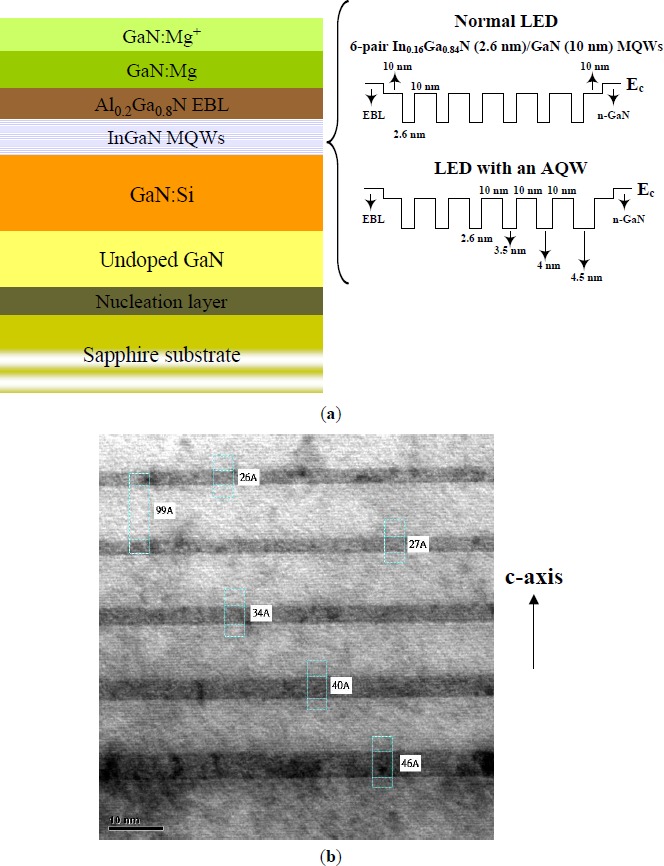
(**a**) Schematic cross-sectional diagram of the epitaxial structures. In comparison with normal LEDs which have a six-pair In_0.16_Ga_0.84_N (2.6 nm)/GaN (10 nm) multiple-quantum-well (MQW), the proposed LEDs feature a modified MQW in which three InGaN wells adjacent to the n-GaN have a graded thickness while the remaining structure is identical to those of normal LEDs; (**b**) High-resolution transmission electron microscopy (HRTEM) image of the modified MQWs in the LED with an asymmetric quantum well (AQW).

As shown in Figure 6a, the LED structures were grown on c-plane sapphire substrates using an atmospheric-pressure MOVPE system (SR2000, Taiyo Nippon Sanso, Japan). During the growth process, precursors of trimethylgallium (TMGa), trimethylindium (TMIn), and NH_3_ were respectively used as the Ga, In, and N sources, while biscyclopentadienyl magnesium (CP_2_Mg) and SiH_4_ were respectively used as the p- and n-type doping sources. Prior to the growth of the InGaN MQWs, a 25-nm-thick low-temperature (560 °C) GaN layer was first deposited, followed by the growth of a 2.5-μm-thick undoped GaN and then a 3-μm-thick Si-doped n-GaN. InGaN/GaN MQWs with a six In_0.16_Ga_0.84_N quantum well separated by 10 nm GaN barriers were used in normal LEDs for 455 nm emissions. We propose a modified MQW in which quantum wells of different thicknesses are used as the active layers of InGaN LEDs. The respective growth temperatures for the wells and barriers of each LED were 800 and 850 °C. Figure 6b shows the high-resolution transmission electron microscopy (HRTEM) image of the MQW active layer of the LED with an AQW. Three quantum wells adjacent to the n-GaN were grown with a graded width, *i.e.*, 3.4, 4.0, and 4.6 nm for the first three quantum wells with reduced thicknesses along the *c*-axis, while the remaining structure is identical to those of normal LEDs. The use of such modified epi-structures will derive benefits from the reduced transport probability of injection carriers (electrons) between the wide well and the narrow well as they are injected from the n-GaN to the AQW [13]. This will redress the insufficient recombination of the electrons with the holes within the InGaN MQWs, and thus help to alleviate the nonradiative Auger recombination and prevent the energetic electrons from escaping from the MQWs. In contrast to the reports in [30,36], the presence of multicolor emissions observed in the LEDs fabricated with an all graded-thickness well could also be suppressed because light emission mostly occurred at the top quantum wells with the indium content and well width identical to those of normal LEDs. A 20-nm-thick p-Al_0.2_Ga_0.8_N electron blocking layer (EBL) was then incorporated on top of the InGaN MQW region. Deposition of the Mg-doped GaN layer terminated the layer stacks. The device process of the InGaN LEDs starts with the deposition of an indium tin oxide (ITO) onto the whole wafer surface to act as the transparent conduction layer (TCL). The wafer was then etched through the active layer down to the n-type GaN to form a 300 × 300 μm^2^ mesa. Finally, a Cr/Au layer was thermally evaporated onto the exposed n-GaN layer along with part of the TCL to serve as the n (p) electrode.

High-resolution X-ray diffraction (HRXRD) (PANalytical, Almelo, The Netherlands) was used to characterize the material quality and structural parameters of the as-grown samples. During temperature-dependent photoluminescence (PL) measurements, the sample was excited using a 30-mW continuous-wave (cw) He-Cd laser (λ = 325 nm) and placed in an Oxford cryostat to vary the operating temperature between 50 and 300 K. The luminescence spectra observed from the samples were dispersed using a monochromator (Acton Research Corporation, Acton, MA, USA) and then detected by a photon multiplier (Acton Research Corporation, Acton, MA, USA), employing a standard lock-in technique. In on-wafer measurements, the completed LEDs without packaging were driven by a Keithley Model 2400 source meter (Keithley Instruments Inc., Cleveland, OH, USA) and the corresponding light output power was measured by a calibrated Newport integrating sphere with a Si detector connected to an optical multimeter (Newport Corporation, Irvine, CA, USA).

## 4. Conclusions

In summary, we have demonstrated the fabrication and characterization of InGaN LEDs with an asymmetric quantum well (AQW). Experimentally, three wells near the n-GaN layer were grown with a graded width to increase the uniformity of carrier distribution within the quantum wells responsible for light emission. For LEDs with an AQW, HRTEM analysis shows the crystalline structure and interface quality remain largely unchanged even if the wide well-containing MQWs were used. Because the epitaxial growth of InGaN/GaN MQWs is fully coherent to GaN, the slight increase in the carrier localization degree of the LED with an AQW may result from variations of the indium distribution within the wide wells caused by the composition pulling effect. However, such an improvement is insufficient for injected carriers to be recombined radiatively at these localized states so that all fabricated LEDs exhibit similar light output performance at low current levels. The peak intensity shifted towards higher current levels, and the maximum light output power of the LED with an AQW is also superior, *i.e.*, 14.97 mW at 580 mA and 13.88 mW at 500 mA, respectively, for the LEDs with and without an AQW. For the LED with an AQW, the light intensity degraded at elevated current levels, which could be attributed to reduced tunneling probability for electrons across the quantum wells with different thicknesses, thus inhibiting the preference of injected carriers to accumulate at the topmost quantum well nearest the p-GaN. Finally, light emissions generated from the wide wells were not observed in the LED with an AQW. This may be due to insufficient carrier density, especially for the holes, within the AQW regions. In addition, the impact of QCSE becomes relatively strong in the wide wells, which will significantly reduce the spontaneous emission rate.
